# Long-Term Aerobic Training Improves Mitochondrial and Antioxidant Function in the Liver of Wistar Rats Preventing Hepatic Age-Related Function Decline

**DOI:** 10.3390/biology11121750

**Published:** 2022-11-30

**Authors:** Mónica Garcia Silva, Paulo Nunes, Paula Oliveira, Rita Ferreira, Margarida Fardilha, Daniel Moreira-Gonçalves, José Alberto Duarte, Maria Manuel Oliveira, Francisco Peixoto

**Affiliations:** 1 Centro de Química de Vila Real (CQVR), Chemistry Department, University of Trás-os-Montes and Alto Douro, 5000-801 Vila Real, Portugal; 2 Instituto de Inovação, Capacitação e Sustentabilidade da Produção Agro-Alimentar (INOV4AGRO), Centro de Investigação e Tecnologias Agroambientais e Biológicas (CITAB), Department of Veterinary Sciences, University of Trás-os-Montes and Alto Douro, 5000-801 Vila Real, Portugal; 3 Labratório Associado para a Química Verde-Rede de Química e Tecnologia (LAQV-REQUIMTE), Department of Chemistry, University of Aveiro, 3810-193 Aveiro, Portugal; 4 Laboratory of Signal Transduction, Institute for Research in Biomedicine, Medical Sciences Department, University of Aveiro, 5000-801 Vila Real, Portugal; 5 Centro de Investigação de Atividade Física, Saúde e Lazer (CIAFEL), Faculty of Sports, University of Porto, 4099-002 Porto, Portugal; 6 Centro de Química de Vila Real (CQVR), Biology and Environment Department, University of Trás-os-Montes and Alto Douro, 5000-801 Vila Real, Portugal

**Keywords:** biochemistry, aerobic training, mitochondria, antioxidant enzymes, oxidative stress, ageing, liver

## Abstract

**Simple Summary:**

This study shows the lifelong benefits of aerobic exercise in preventing early age-related consequences on mitochondrial liver function and antioxidant activity. Although exercise intensity is often cited as crucial for developing adaptive responses in the body, we demonstrated that the duration of the training protocol might be more critical for the adaptive response. Thus, physical exercise prescribers should promote the awareness of physical exercise practitioners about the importance of performing this practice regularly, as the benefits go far beyond those already known in cardiac and skeletal muscle.

**Abstract:**

Most studies on the effects of physical exercise have focused on its influence on muscle tissue, forgetting its interference in liver function. Ageing leads to the progressive impairment of hepatic functions. Several biochemical and bioenergetics parameters were determined to test the impact of a lifelong aerobic training program in the hepatic age-related and the development of an adaptative response. Liver samples were collected from 28 male Wistar rats (4-week-old, 159.4 ± 11.9 g at the beginning of the protocol), randomly distributed into two groups: non-exercised or exercised and submitted to a treadmill exercise program (60 min/day, 5 days/week, at 70% of maximal running speed), for 24 (*n* = 9) or 54 weeks (*n* = 10). A maximal running speed test was performed to determine the training speed. Antioxidant enzyme activity, cellular redox status, oxidative stress, mitochondrial respiratory chain enzymes and respiratory activity were performed in liver samples. Lifelong exercise decreased the age-associated decline in mitochondrial dysfunction, increasing the respiratory rate in state 2 (mitochondrial respiration stimulated by the substrate in the absence of added ADP) (*p* = 0.03) and citrate synthase enzymatic activity (*p* = 0.007). Complex II (*p* < 0.0001) and IV (*p* < 0.001) showed a decrease in enzymatic activity. Ageing-related oxidative stress was also attenuated by physical exercise, as showed by the increase in first-line defense antioxidant enzymes (superoxide dismutase (*p* = 0.07) and catalase (*p* = 0.03)), decreased lipid peroxidation levels (*p* = 0.864 for total fraction, *p* = 0,27 for mitochondrial fraction) and higher glutathione reduced/oxidized ratio (*p* = 0.02). According to our results, the regular practice of exercise can prevent the liver’s mitochondrial dysfunction and loss of antioxidant system efficacy that may arise from ageing, highlighting the benefit of lifelong aerobic exercise in preventing age-related hepatic impairment and associated diseases.

## 1. Introduction

Ageing is characterized by the normal and progressive decline of various physiologic functions leading to a decrease in the ability of the whole organism’s capacity response [1]. From all biological theories of ageing, the mitochondrial theory [2], based on Harman’s free radical theory of ageing [3] is one of the most accepted since it offers one of the best mechanisms to elucidate the degenerative processes as well as other age-related alterations, as age-related diseases [4]. This theory considers the implication of mitochondria as producers and targets of reactive oxygen species (ROS). The accumulation of oxidative damage provoked by this species leads, in part, to mitochondrial dysfunction.

Electron transport chain (ETC) dysfunction and loss of mitochondrial content are some of the mitochondrial abnormalities accompanying ageing. Complex I and IV are the most susceptible enzymatic complexes in ETC to suffer age-related damage, which makes them recognized biomarkers of ageing [5]. These changes can lead to a loss of oxidative phosphorylation (OXPHOS) efficiency, characterized by a lower coupling between the two reactions comprising the OXPHOS process: ETC and ADP phosphorylation. This translates as a decrease in state 3 respiratory rate (mitochondrial respiration initiated by adding ADP in the presence of substrate) and an increase in state 2 (mitochondrial respiration stimulated by the substrate in the absence of and added ADP), associated with a higher ROS production. The increase in ROS production, not accompanied by a response from the scavenging system, results in the exacerbation of oxidative damage [6].

The reduction in or prevention of the accumulated oxidized molecules has been one of the strategies used to attenuate this process, and exercise has emerged as an effective approach capable of preventing it [7]. Many studies have shown that moderate exercise can decrease the incidence of some age-related pathologies, especially those related to oxidative stress [8,9,10]. 

Regular physical exercise is associated with a significant improvement in health quality [11,12]. ROS produced during exercise training are the major factors responsible for the adaptative response [7,13]. This response follows a hormesis curve, where moderate and regular doses of ROS are associated with an increased physiologic function [13]. The intensity of exercise is considered the main exercise parameter responsible for the amount of ROS production. Moderate ROS levels seem to correlate with moderate exercise intensity [14]. Indeed, physical activity guidelines for active ageing recommend exercising at a moderate-to-vigorous intensity to maximize health benefits [9]. However, it remains to be studied if long-term exercising, e.g., through life, always at moderate intensity, which means that intensity is continuously being adjusted to keep disturbing the organisms and imposing the same relative work demand, with the training program as the only variable parameter, can influence exercise-induced adaptations positively or negatively [15].

The stimulation of the antioxidant muscle system, characterized by the modulation of antioxidant enzyme expression, is one of the central chronic adaptations provoked by training programs [9]. This stimulation results in a long-term increase in enzymatic activity, allowing higher effectiveness in ROS scavenging in states of severe oxidative stress, preventing oxidative damage, as shown by the lower levels of lipidic peroxidation, a biomarker of cellular damage, and a higher glutathione reduced (GSH) and glutathione oxidized (GSSG) ratio, correlated with low oxidative damage, typically observed as a response to training [16,17].

Typically, this modulation is an outcome of long-term training protocols. Short-term exercise seems unable to induce a long-term adaptation, even though reports show alterations in the antioxidant system [18].

Recently, the improvement in mitochondria function was pointed out as an outcome of training programs. According to mitohormesis theory, training acts as a stimulus to mitochondria leading to the emission of potential signals, such as ROS or other mitochondrial metabolites, capable of activating metabolic pathways responsible for the improvement of mitochondrial bioenergetic efficiency observed with physical exercise [19,20]. Several studies have demonstrated that both short- and long-term exercise can benefit mitochondrial function [21,22]. However, regarding short-term exercises, a positive outcome is not always observed [23,24].

Nonetheless, it should be noted that the aforementioned outcomes of both the antioxidant system and mitochondrial function depend not only on the duration of the exercise but also on the type of exercise and the intensity, which may vary from individual to individual, justifying the different observations described in the literature [24].

Higher efficiency of OXPHOS is one of the mitochondrial improvements correlated with aerobic training. Training leads to a higher demand for ATP in skeletal muscle, increasing the mitochondria number in the respiratory state 3 [5]. Efficient electron transport between the mitochondrial enzymatic complexes, achieved through increased enzymatic activity, is essential for OXPHOS efficiency. This increase is considered one of the leading mitochondrial improvements correlated with long-term training [25]. 

All the aforementioned modifications are well established in the skeletal muscle [9,26]. However, the multifaceted nature of physical exercise results in a connected response from several organs, such as the heart, liver and brain, as a response to the energy demand of contracting the skeletal muscle during the activity [27]. Thus, it is reasonable to expect that these organs undergo a similar adaptive process as observed in skeletal muscle. Many reports found similar adaptive responses, but the results are inconsistent given the variability of exercise protocols applied and the lack of information regarding the full response of these organs to the different exercise parameters [5,28,29]. 

The liver is one of the organs more susceptible to exercise adaptation due to its central role in providing energy to skeletal muscle during this physical activity [30]. Some reports showed an improvement in the liver’s antioxidant and mitochondrial function during physical exercise [5,31]. However, there is a lack of information concerning which training protocol and which exercise parameters are adequate for improving antioxidant and mitochondrial function, attenuating the consequences of the liver’s ageing. Longer training programs seem to be required for the development of similar adaptative mechanisms to the ones observed in skeletal muscle [25,29]. 

Thus, this study aimed to evaluate the impact of a lifelong training program in rat ageing and the development of an adaptive response by the liver. For that, rats were subjected to a lifetime-long aerobic training program until different ages: 32 weeks old (24 weeks after beginning the exercise protocol) and 62 weeks old (54 weeks after beginning the exercise protocol). To test our hypothesis, we performed the evaluation of several biochemical parameters, such as oxidative stress markers, antioxidant enzyme activities, and bioenergetics parameters, such as mitochondrial complexes activities, mitochondrial mass and mitochondrial respiratory capacity.

## 2. Materials and Methods

### 2.1. Animals and Experimental Groups

Forty male Wistar Unilever rats (4 weeks old) acquired from Charles River Laboratories (France), were used for this study. Animals were housed in polycarbonate cages (5 per cage) (Eurostandart, Type II 1264C, Tecniplast, Italy) and maintained in climate-controlled animal facilities (temperature of 23 ± 2 °C, the humidity of 50 ± 10%, day/night cycle of 12 h) and fed with a standard laboratory diet (Diet Standard 4RF21^®^, Ultragene, Italy). Water and food were provided *ad libitum*. Animals were weighed weekly and the ponderal homogeneity index (PH = 2W_i_/(W_i_ + W_h_)) and ponderal gain (PG = W_2_ − W_1_/W_2_ × 100) were determined, with W_i_ being the lowest animal weight, W_h_ the highest animal weight, W_1_ the initial body weight and W_2_ the final body weight. After 7 days of quarantine and acclimation to the animal facilities, these animals were randomly assigned to one of four experimental groups (ten animals/group, *n* = 10): NEX24 (non-exercising young, sacrificed at 24 weeks after starting the experimental protocol), EX24 (exercise young, sacrifice at 24 weeks of training), NEX54 (non-exercising middle age, sacrificed at 54 weeks after starting the experimental protocol) and EX54 (exercise middle-age, sacrifice at 54 weeks of training). The physical exercise protocol started at 8 weeks of age.

### 2.2. Exercise Training Program

The aerobic training program consisted of running on a treadmill (*Treadmill Control LE 8710*, Harvard Apparatus, Holliston, MA, USA), for 60 min/day, 5 days/week, at 70% of maximal running speed, during 24 or 54 weeks. The inclination of the treadmill was maintained at 0° through all exercise protocol. A maximal running speed test was performed to determine the training speed. After warming up for 5 min at 15 m/min, treadmill speed was increased 5 m/min every 1 min, until interruption (unable/unwilling to maintain pace with the treadmill belt, despite reinforcement). Maximal running speed was defined as the last speed supported for an entire minute. In the first two weeks of adaptation to the exercise protocol, the intensity and duration were progressively increased until the target speed and duration were reached. During the training program, the absolute intensity (running speed) was adjusted according to a maximal running speed test performed every 6 weeks, maintaining the relative intensity by 70% of the maximal running speed.

The non-exercising groups were regularly placed on a non-moving treadmill for a couple of minutes to be subjected to the same handling stress as the animals belonging to the exercised groups.

### 2.3. Sample Collection

**Figure 1:**
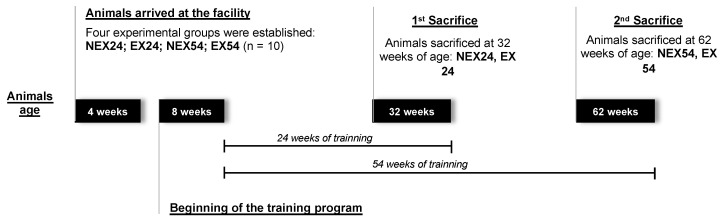
Experimental design.

Animals were euthanized by an intraperitoneal injection of ketamine (Imalgene^®^ 1000, Merial S.A.S, Lyon, France) and xylazine (Rompum^®^, Bayer Healthcare S.A., Kiel, Germany) overdose, preceded by their exsanguination via intracardiac punction. The sacrifice occurred at two different times: at the 24th week of training, when the animals were 32 weeks old (young) and at the 54th week of training when the animals were 62 weeks old (middle-age). The experimental protocol can be observed in Figure 1. These moments corresponded to different stages of animals’ age, which allowed the evaluation of the training effects during ageing.

During the animals’ sacrifice, the livers were collected, weighed and divided into several portions before being frozen in liquid nitrogen and stored at −80 °C for posterior oxidative stress assessment and mitochondrial bioenergetics evaluation.

### 2.4. Oxidative Stress Evaluation

*Liver homogenization:* A small portion of the frozen livers was weighed (approximately 1 g), added to 20% (*w*/*v*) ice-cold phosphate buffer (50 mM, pH 7.0), homogenized and sonicated for 2 min. The homogenate was subjected to three centrifugation cycles, at 4 °C: 1500× *g* for 10 min, 8000× *g* for 10 min and 14,000× *g* for 10 min. The protein supernatant obtained in the last centrifugation was used to measure antioxidant enzyme activities and to determine the reduced glutathione (GSH) and oxidized glutathione (GSSG) ratio. The lipid peroxidation (LOP) was evaluated using the pellets obtained from the total and mitochondrial fraction corresponding to those obtained during the first centrifugation cycle, as well as in the second and third cycles, respectively. The total protein content of the supernatant and pellets obtained was evaluated by the Biuret method [32], using bovine serum albumine (BSA) as standard.

*Antioxidant enzymes activities*: All antioxidant enzymatic activities evaluation assays were carried out at 30 °C. Superoxide dismutase (SOD) activity was evaluated spectrophotometrically, at 560 nm, by the method described by Payá [33], with slight modifications [34], using the xanthine-xanthine oxidase system. Assays were performed in the presence of a potassium phosphate buffer (50 mM, pH 7.4), Ethylenediamine tetraacetic acid (EDTA, 1 mM), hypoxanthine (10 mM), nitroblue tetrazolium chloride (NBT, 10 mM) and approximately 0.4 mg protein of enzymatic extract. The kinetic was initiated by the addition of xanthine oxidase. Results were expressed as U.min^−1^.mg^−1^ protein. One unit of SOD was reported as the amount of SOD inhibiting the reduction rate of NBT to formazan by 50%. Catalase (CAT) was assayed polarographically using a Clark-type oxygen electrode as described by del Río [35], with minor modifications [36], in the presence of a potassium phosphate buffer (50 mM, pH 7.0) and hydrogen peroxide (8.82 M). The reaction was initiated with the addition of 0.4 mg protein of samples to the mixture. CAT activity was presented in mmol H_2_O_2_ consumed.min^−1^.mg^−1^ protein. Glutathione peroxidase (GPx) activity was evaluated spectrophotometrically, at 340 nm, by the method of Mannervik [37] with slight modifications [36]. The activity was measured indirectly by determining the nicotinamide-adenine dinucleotide phosphate (NADPH) (ε = 6.22 × 10^3^ M^−1^.cm^−1^) oxidation by the action of glutathione reductase. The reaction mixture contained a potassium phosphate buffer (100 mM pH 7.0), EDTA (1 mM), reduced glutathione (GSH, 100 mM), glutathione reductase, NADPH (10 mM) and 0.4 mg protein of enzymatic extract. The reaction was started with the addition of *t*-butylhydroperoxide (7.7 M) to the system. The results were expressed as μmol NADPH oxidized.min^−1^.mg^−1^ protein. Glutathione reductase (GR) was measured according to Carlberg and Mannervik [38] with minor modifications [39], under magnetic stirring. Assays were performed spectrophotometrically at 340 nm. The reaction system consisted of a potassium phosphate buffer (100 mM, pH 7.4), EDTA (0.5 mM), NADPH (10 mM) and 1.5 mg protein of enzymatic extract. The addition of oxidized glutathione (GSSG, 100 mM) to the system was responsible for the start of the kinetic reaction. The GR activity was expressed as μmol NADPH oxidized.min^−1^.mg^−1^ protein.

*Lipid Peroxidation:* Nonspecific lipid peroxidation (LOP) levels were evaluated by measuring the levels of lipid peroxides as the amount of thiobarbituric acid reactive substances (TBARS) formed, as described by Ottolenghi [40], with some modifications. The hepatic lipid extract was mixed with 1 mL of thiobarbituric acid (TBA) reagent (0.38% (*m*/*v*) TBA, 37.5% (*m*/*v*) trichloroacetic acid (TCA) and 0.015% (*m*/*v*) butylated hydroxytoluene (BHT)), heated at 100 °C for 15 min and cooled down by ice immersion. The mixture was centrifuged at 1600× *g* for 10 min, at 4 °C, and the supernatant was collected to perform the assay. Lipid peroxidation was estimated spectrophotometrically, at 532 nm, by the appearance of malondialdehyde (MDA). Results were expressed as μmol MDA.mg^−1^ protein using molar extinction coefficient of 1.56 × 10^5^ M^−1^ cm^−1^.

*GSH and GSSG Ratio:* The ratio of reduced GSH and oxidized GSH (GSSG) was evaluated fluorometrically, according to Hissin and Hilf [41], using the fluorochrome *orto*-phthalaldehyde (OPT) excitation at 339 nm and emission at 426 nm. To determinate GSH content, samples were incubated in the dark, for 15 min, in a potassium phosphate buffer (100 mM, pH 8.0), EDTA (5 mM) and OPT (200 μL, 1 mg/mL ethanol). To measure GSSG levels, samples were incubated at room temperature with *N*-ethylmaleimide (NEM, 40 mM), for 30 min and more 15 min in the dark after the addition of sodium hydroxide (100 mM, pH 12.0) and OPT (200 μL, 1 mg/mL ethanol). Concentrations were obtained using standard curves made with different concentrations of GSH and GSSG, and results were expressed as their ratio (GSH/GSSG).

### 2.5. Mitochondrial Bioenergetics Evaluation

*Isolation of rat liver mitochondrial fraction*: The isolation of liver mitochondrial fraction was performed by differential centrifugation, according to Peixoto [42], with minor modifications. A small portion of frozen liver was weighed (±1 g), added to an ice-cold homogenization medium (200 mM sucrose, 10 mM tris-HCl, 1 mM EDTA, pH 7.4) and homogenized. The mixture was centrifugated at 800× *g*, for 10 min, at 4 °C. Mitochondria were obtained from supernatant centrifugation at 14,000× *g* for 10 min at 4 °C. The mitochondrial fraction sedimented was washed once and resuspended in homogenization medium at a protein concentration of 20–30 mg/mL. Total protein concentration was evaluated by the Biuret method [32], using BSA as the standard.

*Mitochondrial enzymatic activities:* The mitochondrial fractions were submitted to three cycles of freezing–thawing to disrupt intact mitochondria. All the enzymatic activities were monitored spectrophotometrically at 30 °C. Complex I (NADH dehydrogenase) activity was measured at 340 nm, under magnetic stirring, following the oxidation of nicotinamide adenine dinucleotide (NADH) (ε = 6.22 × 10^3^ M^−1^.cm^−1^), according to Félix [36], with slight alterations, in a medium containing a phosphate buffer (50 mM, pH 7.6), bovine serum albumin (BSA, 50 mg/mL), decylubiquinone (50 mM), triton X-100 (10% (*m/v*)) and 0.2 mg protein of mitochondrial fraction. The reaction started with the addition of NADH (10 mM) to the system. Results were expressed as μmol NADH oxidized min^−1^ mg^−1^ protein. Only rotenone sensitive activity was considered. Complex II (succinate dehydrogenase) was evaluated at 600 nm by the reduction of 2,6-dichlorophenolindophenol (DCPIP) (ε = 2.1 × 10^4^ M^−1^.cm^−1^), as described by Peixoto [34]. The reaction was started by the addition of decylubiquinone (50 mM) to a mixture containing a phosphate buffer (100 mM, pH 7.6), BSA (50 mg/mL), rotenone (1 mg/mL), KCN (100 mM), ATP (10 mM), succinate (1 M), 2,6-dichlorophenolindophenol (DCPIP, 5 mM) and 0.2 mg protein of mitochondrial fraction. Complex II activity was presented as μmol DCPIP reduced min^−1^ mg^−1^ protein. Complex IV (cytochrome *c* oxidase) was determined by the oxidation of reduced ferrocytochrome *c* (100 μM) (ε = 19 × 10^3^ M^−1^.cm^−1^) prepared in a phosphate buffer (50 mM, pH 7.6) as previously described [36]. Results were presented as μmol cytochrome *c* oxidized min^−1^ mg^−1^ protein. Activities were normalized to citrate synthase (CS) activity which was evaluated following the reduction of 5,5′-dithiobis(2-nitrobenzoic acid) (DTNB) (ε = 1.36 × 10^3^ M^−1^.cm^−1^), at 412 nm in a buffer containing Tris-HCl (200 mM, pH 8.0), DTNB (1.01 mM), triton X-100 (0.02% (*m*/*v*)), oxaloacetate (10 mM) and acetil-CoA (0.037 mM), as described by Félix [36]. The kinetic reaction was initiated with the addition of 0.013 mg protein of mitochondrial extract. Results were expressed as mmol TNB min^−1^ mg^−1^ protein.

### 2.6. Mitochondrial Respiratory Activity

*Tissue preparation:* Approximately 50 mg of frozen liver was weighed, added to ice-cold 0.2% (*m*/*v*) of respiratory medium (220 mM mannitol, 75 mM sucrose, 0.5 mM ethyleneglycol- bis(β-aminoethyl)-N,N,N′,N′-tetraacetic acid (EGTA), 3 mM MgCl_2_, 10 mM KH_2_PO_4_, 0.1% (*m*/*v*) BSA, pH 7.6) and then homogenized for a short period of time (maximum 10 cycles). Samples were kept in ice until further evaluation.

*High-resolution respirometry:* Mitochondrial oxygen consumption rate was assessed at 30 °C, with magnetic stirring, using an oxygraph-2k high-resolution respirometer (OROBOROS^®^ Instruments; Innsbruck, Austria). Assays started with the addition of 2 mL of tissue preparation (2 mg tissue/mL respiratory medium) to each chamber of the oxygraph and incubated during 15 min, for basal respiratory assessment, before the addition of a respiratory substrate. Oxygen flux was measured by mitochondria energization with complex I substrate, pyruvate/malate (10/5 mM), and complex II substrate, and succinate (20 mM) in the absence of ADP for the evaluation of state 2 respiration (leak respiration). Rotenone (0.5 μM) and antimycin A (5 μM) were added to inhibit the respiration supported by substrates for complexes I and II, and to allow the determination of the specific respiration of complexes I and II. Mitochondrial respiration was measured and analyzed by the Datlab 4 software (version 4.2.1.50, OROBOROS^®^ Instruments) and the results were expressed as pmol O_2_ min^−1^ mg^−1^ liver.

### 2.7. Statistical Analysis

The results were expressed as mean ± SD (standard deviation) of at least four independent experiments. All data were imported to GraphPad Prism^®^ (GraphPad Software, version 6.0, Inc., San Diego, CA, USA). The normality of distribution was checked with the Shapiro–Wilk test. The organs’ mean relative weight was determined (weight of the organ/final body weight of each animal). Statistical significance between experimental groups was tested using a Two-way ANOVA followed by Tukeys’s Multiple Comparison Test (two-tailed). A value of *p* < 0.05 was considered statistically significant.

## 3. Results

### 3.1. Mortality Rate, Body Weight and Food and Water Consumption

In order to explore the effect of age and exercise on male rats, we measured the body weight, food and water consumption weekly to minimize animal stress. This assessment helps us to understand the general condition of each animal.

Table 1 presents the ponderal homogeneity index (PH), mortality rate, mean initial and final animals’ body weight, ponderal gain (PG) and mean relative liver weight. The PH is an indicator of the initial homogeneity of the experimental groups. Animals from the control groups (NEX24 and NEX54) showed the highest PH variation (0.63), and the exercised groups showed the lowest variation (0.55–0.56). While the animals were all acquired at the same age, their body weights differed. Two animals died during the experimental work: one animal from the NEX24 group and the other from the exercised group EX24. Data from these animals were excluded from the study.

At the beginning of the experimental protocol, the mean animals’ body weight was statistically different only for the NEX54 group, presenting the lowest value. Nevertheless, a statistically significant difference was observed at the end of the study in the mean body weight in all exercised groups compared with non-exercised groups (*p* < 0.05). It was observed that the exercised groups had a lower weight than the non-exercised ones, and concomitantly the PG was higher in the non-exercised groups (NEX24 and NEX 54). (*p* < 0.05). As for changes in liver weight, the only statistical difference was the highest mean relative liver weight for the EX24 group (*p* < 0.05). However, the change in the animal’s weight cannot explain this result.

The mean values of food and water consumption at the beginning and the end of the experimental protocol can be observed in Table 2. Although we found two groups (EX54, EX24) with differences concerning the others, these differences did not seem to have any physiological relevance since the values obtained were all within what was expected for the consumption of these animals.

Regarding food consumption, in the first week of the study, no significant difference was observed between groups. Non-exercised animals (NEX24 and NEX54) showed the highest water consumption at the beginning and the lowest intake at the end of the study. The opposite was verified in the exercised groups (EX24 and EX54).

### 3.2. Lifelong Exercise, Chronological Age and Oxidative Stress

#### 3.2.1. Antioxidant Enzymes

Physical exercise is increasingly recognized as beneficial for health and disease prevention. However, initial adaptation to exercise can have different effects on cells, including an increase in the formation of reactive oxygen species and inflammatory mediators that ultimately lead to oxidative stress. This scenario depends on the type and intensity of exercise and the individual’s training status. Therefore, it is crucial to understand how the antioxidant system responds to each type of exercise. Catalase and glutathione peroxidase are two crucial antioxidant enzymes that play an essential role in the breakdown of hydrogen peroxide. Glutathione reductase plays a crucial role in regulating the redox cycle of glutathione, the most relevant endogenous antioxidant in the non-enzymatic antioxidant system.

Table 3 shows the biologic significance of differences between all four experimental groups. The activity of hepatic antioxidant enzymes CAT, GPx and GR were significantly decreased in NEX54 animals, with losses of 50%, 11% and 30%, respectively, compared with NEX24 (*p* < 0.001). On the contrary, SOD activity did not show any decrease concerning ageing (*p* = 0.6). It is known that the expression of some of the antioxidant enzymes is reduced with ageing, and this decrease is organ-dependent, but age cannot explain these differences since they are not old animals.

Exercise for 24 weeks (EX24) resulted in a decrease, with statistical significance, of 15% in GPx activity (*p* = 0.003) and 15% in GR activity (*p* < 0.001) compared to sedentary young animals (NEX24). Contrarily, SOD and CAT increased, but only the stimulation of SOD activity, by 20%, was statistically significant (*p* < 0.001).

Training for 54 weeks (EX54) was able to increase both SOD and CAT activity by 10% and 27%, respectively, compared with NEX54 animals. However, only CAT increased with statistical significance (*p* = 0.03). It is not easy to systematize the effects of physical exercise on enzymatic antioxidant activity, as they vary with the type of exercise and the animal species used. However, our results indicate that after 54 weeks of exercise, the increase in SOD and CAT activities will be sufficient to counterbalance the ROS production induced by physical activity performed on the treadmill.

#### 3.2.2. Biochemical Oxidative Stress Markers

It is well described in the scientific literature that regular aerobic exercise leads to an antioxidant system response and a subsequent decrease in oxidative stress. Oxidative stress causes remarkable changes in biological structures, including cell membranes, lipids, proteins and nucleic acids, and is involved in numerous diseases. Reduced glutathione (GSH) is considered one of the most important scavengers of reactive oxygen species (ROS), and its relationship to oxidized glutathione (GSSG) has been used as a marker of oxidative stress. The end products of lipid peroxidation such as malondialdehyde (MDA) were measured by the thiobarbituric acid reactive substances (TBARS) assay. Table 4 indicates the biological significance of differences in biochemical oxidative stress markers between all four experimental groups. At 62 weeks of age (NEX 54), TBARS products formed in the total and mitochondrial fraction suffered a rise of 91% and 23%, respectively. Nevertheless, only the increase in the total fraction was statistically significant (*p* = 0.007). This result is not surprising, considering that mitochondria are equipped with antioxidant defenses to quench ROS.

Twenty-four weeks of training (EX24) led to an increase in lipid peroxidation by 96% in the total fraction (*p* < 0.001) and by 67% in the mitochondrial fraction (*p* < 0.001), when compared with the NEX24 group. This increase could be explained by the increase in ROS production as a consequence of the exercise program. The absence of a stimulation in antioxidant enzymes activity and the consequence failure to scavenge ROS could lead to the augmentation of lipid peroxidation levels. So, it seems that the ROS produced during the exercise were not sufficient to provoke an adaptative response towards the antioxidant enzymes activity but were enough to cause cellular damage. However, increasing the exercise for 54 weeks (EX54) led to a decrease in the TBARS level, in both lipidic fractions, when compared with non-exercise animals (NEX54). The decrease in MDA content for the group that exercised for longer was certainly due to the increase in the activity of antioxidant enzymes, as a consequence of the lifelong training program and development of adaptative mechanisms as a response to the ROS produced during exercise practice. It seems that 24 weeks of training were not sufficient to induce an adaptation towards the antioxidant system, leading to cellular damage, whereas 54 weeks of training were capable to induce adaptative mechanisms and, consequently, decrease cellular damage. GSH and GSSG ratio was neither affected by ageing nor by training for 24 weeks. However, 54 weeks of training were able to increase GSH/GSSG, compared with young animals untrained (NEX24) and trained young animals (EX24) by 35% and 45%, respectively (*p* = 0.02). This increase in the GSH/GSSG ratio resulting from physical exercise shows that the exercise performed is beneficial and that performing physical exercise for longer periods gives even better results.

#### 3.2.3. Lifelong Exercise, Chronological Age and Mitochondrial Bioenergetics

**Figure 2:**
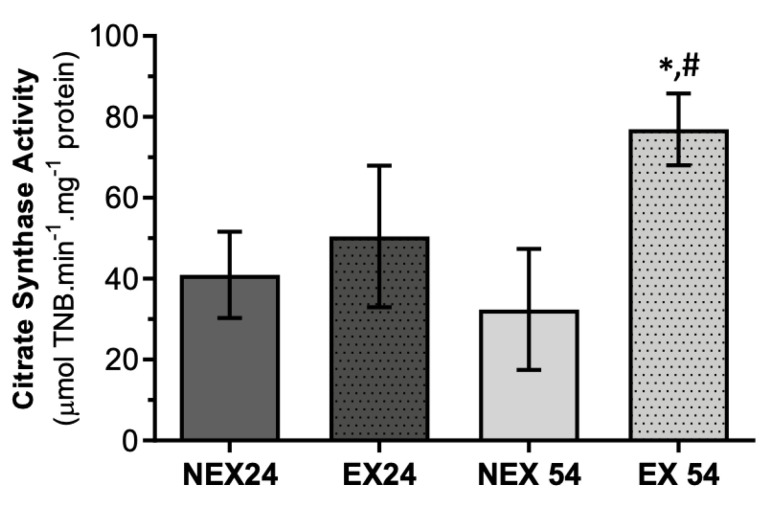
Effect of ageing and training in Citrate synthase activity. Values are means ± SD (*n* = 7), with two replicates. * *p* < 0.05 compared with NEX24; # *p* < 0.05 compared with NEX54; NEX24 (non-exercising young, sacrifice at 24 weeks of training), EX24 (exercise young, sacrifice at 24 weeks of training), NEX54 (non-exercising middle-age, sacrifice at 54 weeks of training), EX54 (exercise middle-age, sacrifice at 54 weeks of training).

Citrate synthase (CS) levels are routinely used as a marker of mitochondrial content [43]. All respiratory complexes’ activities were normalized to citrate synthase activity, a biomarker of mitochondrial mass, because only then would we be able to measure the specific activity of the different complexes without being affected by the mitochondrial content. The activity of CS (Figure 2) was found to decrease with age in NEX54 animals by 21%. We know there is a trend towards a decrease in CS activity in old mice compared to young mice, which may explain the trend observed in the NEX24 group towards NEX54.

The exercise increased CS activity in both ages tested. However, the result was only significant in EX54 animals (58%) compared with NEX54.

Training for 24 or 54 weeks was capable of increasing the mitochondrial content. However, only 54 weeks of exercise significantly increased the mitochondrial content (*p* = 0.007) and could reverse the depletory effect observed with age. This result shows that at 24 weeks of exercise, this activity was already increased, thus indicating an increase in mitochondrial content. However, a longer time was necessary to observe a significant increase in mitochondrial content. The mitochondrial biogenesis probably results from an adaptive process to the exercise, but that will be a slower process.

**Figure 3:**
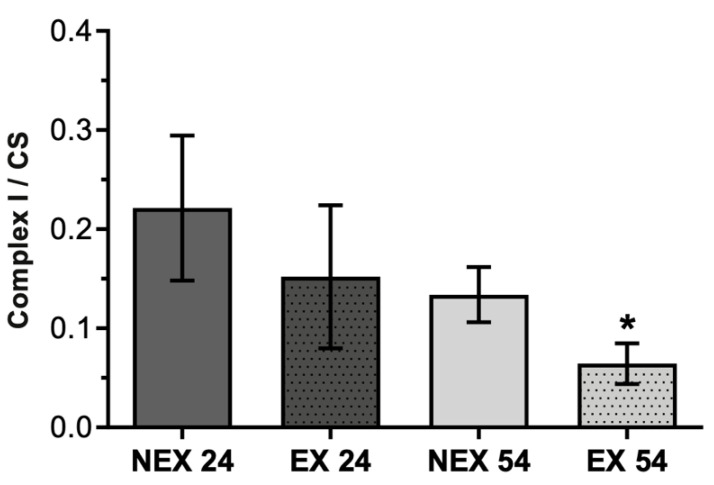
Effect of ageing and training in Complex I/CS. Values are means ± SD (*n* = 7), with two replicates. * *p* < 0.05 compared with NEX24; NEX24 (non-exercising young, sacrifice at 24 weeks of training), EX24 (exercise young, sacrifice at 24 weeks of training), NEX54 (non-exercising middle-age, sacrifice at 54 weeks of training), EX54 (exercise middle-age, sacrifice at 54 weeks of training).

Ageing causes a decrease in complex I activity, which was more evident in the exercised groups, although differences were only evident when comparing the NEX24 and EX54 groups (Figure 3).

NEX54 animals presented a decrease in complex I/CS by 40% compared with young animals (NEX24). Animal training until 32 weeks of age (EX24) diminished the evaluated ratio by 32%. Training until 62 weeks old (EX54) also provoked a statistically significant decrease of 52% compared to young untrained animals (*p* = 0.007).

**Figure 4:**
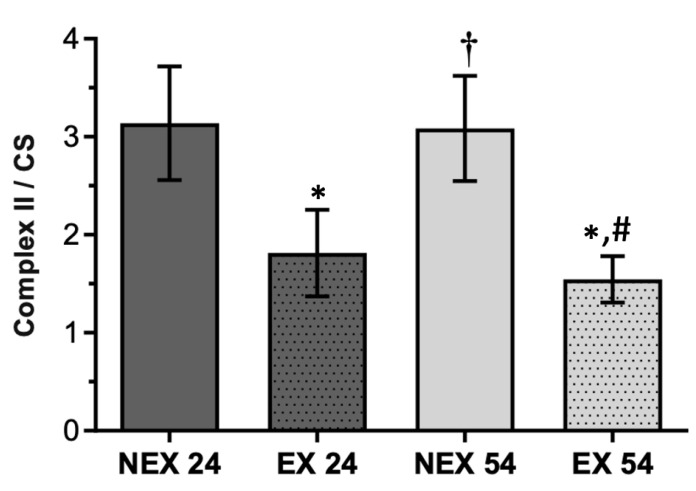
Effect of ageing and training in Complex II/CS. Values are means ± SD (*n* = 7), with two replicates. * *p* < 0.05 compared with NEX24; † *p* < 0.05 compared with EX24; # *p* < 0.05 compared with NEX54; NEX24 (non-exercising young, sacrifice at 24 weeks of training), EX24 (exercise young, sacrifice at 24 weeks of training), NEX54 (non-exercising middle-age, sacrifice at 54 weeks of training), EX54 (exercise middle-age, sacrifice at 54 weeks of training).

For complex II, the ageing process did not appear to have any significant impact until 62 weeks of age (Figure 4).

Training for 24 (EX24) and 54 (EX54) weeks showed a similar effect leading to a decrease in complex II enzymatic activity, when normalized by CS activity, of 42% and 50%, respectively, when compared with same-aged untrained animals (NEX24, NEX54). Alterations caused by both training durations, 24 and 54 weeks, were statistically significant (*p* < 0.001). From this, we can deduce that physical exercise, even after 24 weeks, is already capable of causing a remodeling in the activity of complex II of the electron transport chain (ETC) and that this effect persists after 54 weeks of physical activity.

**Figure 5:**
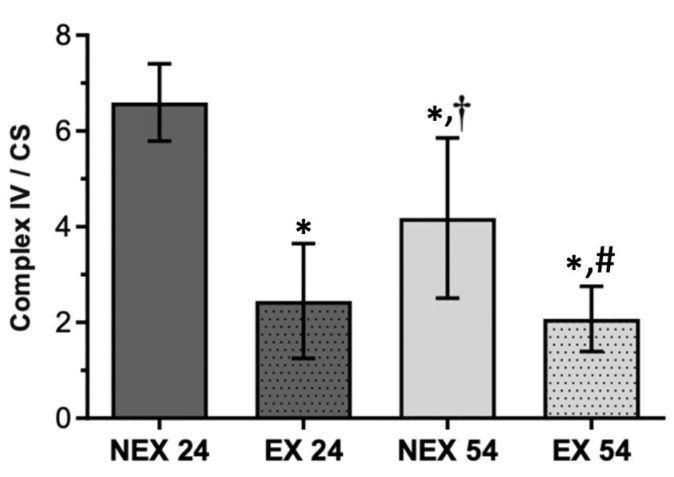
Effect of ageing and training in Complex IV/CS. Values are means ± SD (*n* = 7), with two replicates. * *p* < 0.05 compared with NEX24; † *p* < 0.05 compared with EX24; # *p* < 0.05 compared with NEX54; NEX24 (non-exercising young, sacrifice at 24 weeks of training), EX24 (exercise young, sacrifice at 24 weeks of training), NEX54 (non-exercising middle-age, sacrifice at 54 weeks of training), EX54 (exercise middle-age, sacrifice at 54 weeks of training).

Complex IV/CS (Figure 5) presented a similar response to complex II and CS ratio facing the training program, but not to the ageing process. NEX54 animals exhibited a decrease of 36% in complex IV activity, normalized by CS activity, compared to young animals (NEX24). Twenty-four weeks of exercise (EX24) also resulted in a 63% diminution of complex IV/CS. Fifty-four weeks of training had a similar effect than 24 weeks of exercise once it decreased complex IV/CS by 50%.

In complex IV/CS, as observed in complex II/CS, the impact of both training durations, 24 and 54 weeks, were statistically significant (*p* < 0.001), when compared with non-exercised groups. The decreased activity of the complexes may have resulted from an increase in their efficiency, thus contributing to the decrease in the production of ROS.

#### 3.2.4. Lifelong Exercise, Chronological Age and Mitochondrial Respiratory Capacity

**Figure 6:**
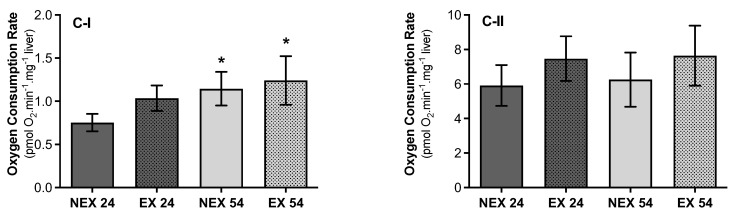
Effect of ageing and training in mitochondrial state 2 respiratory rates: Mitochondrial respiration measured, in liver homogenate, with specific substrates for complex I (**C-I**) and complex II (**C*-*II**). Oxygen consumption rates were measured after the adding of 10 mM pyruvate and 5 mM malate (**C-I**), and oxygen consumption was obtained with 10 mM succinate (**C-II**). Rotenone (0.5 μM) and antimycin A (5 μM) were added to determine the specific respiration of complexes I and II, as described in the Material and Methods section. Values are means ± SD (*n* = 7), with two replicates. * *p* < 0.05 compared with NEX24; NEX24 (non-exercise young, sacrifice at 24 weeks of training), EX24 (exercise young, sacrifice at 24 weeks of training), NEX54 (non-exercise middle-age, sacrifice at 54 weeks of training), EX54 (exercise middle-age, sacrifice at 54 weeks of training).

Ageing and exercise had a similar effect in state 2 respiratory rate supported by complex I substrates. Pyruvate + malate mixture activates mitochondrial dehydrogenases by reducing nicotinamide adenine dinucleotide and feeding electrons into complex I, resulting in a down thermodynamic cascade through the Q-cycle and Complex III of the electron transport system to Complex IV and O_2_. So, using the substrate combination of pyruvate + malate (Figure 6C-I), NEX54 animals showed an increase in the respiratory rate (34%), taking young animals as a reference (NEX24). Exercise for 24 weeks (EX24) led to the augmentation of oxygen consumption by 27% compared to non-exercise young animals (NEX24) (*p* = 0.03). Exercise for 54 weeks (EX54) had a comparable effect once it increased by 8% of the state 2 respiratory rate, but without statistical significance (*p* = 0.4). When mitochondria were energized with succinate, complex II-driven specific substrate, significant differences in oxygen consumption rate, were not found between groups (*p* > 0.05) (Figure 6C-II).

## 4. Discussion

Dysfunctional mitochondria and an ineffective antioxidant system are alterations well described during the ageing process. Training is associated with improved mitochondrial function and the stimulation of the antioxidant system. Thus, physical activity could be a practice capable of attenuating some consequences of the ageing process. Indeed, several reports using old animals subjected to training programs show that exercise can mitigate the ageing consequences, regarding mitochondria and antioxidant function, in several organs and tissues, such as the brain, heart and skeletal muscle [5,31,44]. However, the impact of exercise on liver function and the reversal of liver ageing remains unclear.

In this study, the training protocol was shown to impact animals’ body weight and food/water intake, as expected, whereas the ageing process did not affect these parameters. Exercised animals presented a lower average final weight gain when compared to non-exercised animals (Table 1), which may have resulted from physical exercise. Furthermore, at the end of the assay, the exercised groups ingested higher water content than the non-exercised (Table 2), which is explained by exercise-induced dehydration.

Regarding liver mitochondrial fitness, there was an age-related decline that significantly impacted the liver’s mitochondria bioenergetics. A loss of activity in all mitochondrial enzymatic complexes was related with age increase, being more obvious in complex IV (Figure 5) rather than in complex I (Figure 3) and II (Figure 4). These alterations were also accompanied by a slight reduction in CS activity (Figure 2).

On the other hand, when physical exercise was maintained for 54 weeks, the exercise induced a remodeling in the activity of mitochondrial respiratory complexes (Figure 3, Figure 4 and Figure 5) and reverted the loss of age-related mitochondrial content, characterized by an increase in CS activity (Figure 2). This means that the more regular and long-lasting the exercise, the more health benefits it will have. It is not enough to go to the gym occasionally, and it is necessary to go long-term.

Modifications in mitochondrial biogenesis with ageing have been reported in the literature [29,45,46]. Reports showed a decrease in complex I and IV activities in organs from old rats and a loss of mitochondrial mass in different organs, such as the liver [47]. Other studies support the correlation of the loss of electron transport with the decrease in mitochondria content [48,49]. The results obtained in the oldest animals evaluated in this study (62 weeks) reflected such findings, even if they cannot be considered old animals.

Mitochondrial biogenesis modulation, which is characterized by increased mitochondria mass, is one of the main adaptative changes observed with training.

These changes have been well described in the literature in several tissues and organs [19,46]. Nevertheless, some reports failed to demonstrate an increase in the liver’s mitochondria content in response to training programs with smaller durations, contrary to our results [25,50]. 

There are some inconsistencies in the literature concerning the ageing impact on mitochondrial respiratory capacity. Delaval and collaborators [51] did not observe an alteration in hepatic mitochondrial respiratory rate collected from 94 weeks old Wistar rats when complex-I substrates energized mitochondria. Otherwise, Duicu and collaborators [48] observed a lower mitochondrial respiratory rate in cardiac mitochondria in older rats. The scarce number of studies may justify these contradictory results [51]. In our study, an increase in state 2 respiratory rate was observed for mitochondria energized with malate/glutamate (Figure 6C-I), a complex-I specific substrate. However, no alterations in oxygen flux were observed in middle-aged animals when mitochondria were energized with complex II specific substrate succinate (Figure 6C-II).

Interestingly, an increase in oxygen flux in respiratory state 2 was also observed with training for 24 and 54 weeks, when mitochondria were energized with pyruvate/malate (Figure 6C-I) and succinate (Figure 6C-II).

In the brains of aged mice, exercise can increase mitochondrial respiration supported by complex I substrates, thus affecting ATP production and mitochondrial function [52]. Han and Kim [53] also observed that endurance exercise augments respiration and decreases RCI, indicating an uncoupling of respiration and oxidative phosphorylation. In our work, the increased respiratory rate observed for the exercised groups seemed to result from the increase in the liver’s mitochondrial biogenesis and not from a decreased efficacy of oxidative phosphorylation. Nevertheless, in a different exercise, training significantly increased hepatic mitochondrial respiration observed, and that does not appear to be dependent on increases in mitochondrial content [25]. Although mitochondrial muscle content often increases with training, mitochondrial adaptations are not needed to facilitate maximal oxygen uptake [54].

Mitochondrial dysfunction is frequently correlated to a state of oxidative stress provoked by the observed increase in ROS production [2,3]. The antioxidant system loses its scavenging capacity with ageing, leading to inefficient ROS elimination. These alterations can result in oxidative damage at several cellular levels, including the mitochondria [14].

High levels of peroxidation, a low GSH:GSSG ratio and a loss of antioxidant capacity are described in reports as age-related alterations in different organs [55], and the liver is no exception [56]. Nevertheless, there is controversy regarding the response of different antioxidant enzymes to the ageing process, especially in the liver, as they appear to respond differently to the ageing process. Our results showed that the beginning of the ageing process had a negligible impact on liver antioxidant capacity. Among all the antioxidant enzymes evaluated, only CAT (Table 3) and GR (Table 3) showed a loss of activity with ageing. SOD and GPx activity were not affected (Table 3). This lack of significant alterations is consistent with the absence of changes in the GSH and GSSG ratio (Table 4). However, we observed an increase in lipid peroxidation levels (Table 4) but only in the total lipid fraction isolated from middle-aged animals’ livers. The age of the older animals, 62 weeks old, i.e., middle-aged animals, may justify the absence of significant alterations in some of the mitochondrial and oxidative stress parameters evaluated. Most studies use animals with ages between 96 and 120 weeks, i.e., old animals, which can explain some of the reported differences [57].

In this investigation, 24 weeks of training could not adequately stimulate the hepatic antioxidant system, only leading to stimulation in SOD activity (Table 3). Although this increase may be considered an adaptive mechanism to exercise, we observed high lipid peroxidation levels (Table 4), indicating oxidative damage. The absence of a stimulation in antioxidant enzymes activity and the subsequent failure to scavenge ROS could lead to the augmentation of lipid peroxidation levels. So, it seems that the ROS produced during the exercise were not sufficient to provoke an adaptative response towards the antioxidant enzymes activity but were enough to cause cellular damage. Lipid peroxidation values drops in EX54 (Table 4), were probably, due to the increase in the activity of antioxidant enzymes (SOD and CAT) [58], as a consequence of the lifelong training program and development of adaptative mechanisms as a response to the ROS produced during exercise practice. A higher GSH/GSSG ratio was also observed (Table 4). Maintaining an optimum GSH/GSSG ratio is essential to cell viability. An inappropriate GSH/GSSG ratio induces significant changes in the mechanism of cellular redox-dependent signaling controlled both nonenzymatically and enzymatically. In summary, these results point out that 24 weeks of training were not sufficient to induce an adaptation towards the antioxidant system, leading to cellular damage, whereas 54 weeks of training were capable to induce adaptative mechanisms and, consequently, decrease cellular damage.

In the group that exercised for a longer time (EX54), the fact that the activity of the GR and GPx enzymes was reduced (Table 3) may have contributed to the increase in the GSH/GSSG ratio. This may have resulted from a cellular adaptation that will have led to a greater efficiency of the antioxidant mechanisms and, at the same time, a lowering of the production of superoxide anion and hydrogen peroxide by mitochondria [59]. However, the hepatic antioxidant system response to aerobic training is controversial in the literature. Mallikarjuna and co-workers [60] found an increase in hepatic antioxidant enzymes with a training protocol for eight weeks (treadmill running, 23 m/minute, 30 min/day, five days/week). On the contrary, Yoon and collaborators [61] showed a decrease in the main antioxidant enzymes with a similar training protocol but only for five weeks. The different training programs could explain such inconsistencies and the different responses each antioxidant enzyme appears to have to each exercise parameter, i.e., frequency, duration, and intensity. Nonetheless, according to the literature, the training program duration seems to be one of the parameters essential for developing the liver’s adaptation to exercise, with this adaptation not being linear in time [44,62]. Our results follow this trend, supporting training duration as a crucial parameter for a chronic hepatic adaptation to exercise. Fifty-four weeks of training, considered as long-term exercise, were capable of triggering hepatic antioxidant system chronic adaptation to exercise.

Chronic liver disease, associated with age increase, is a severe and neglected public health problem mainly because it is classified as a silent pathology, and the onset of symptoms is late. Unlike other types of chronic disease, prevention is possible and, when detected early, can even be treated and cured [61]. Due to the crucial role that oxidative stress and mitochondria play in liver disease, it is reasonable to assume that improvements in these parameters may be a rational strategy for the treatment and/or prevention of hepatic pathologies. Furthermore, according to our results, the training program’s duration seems essential for the liver’s adaptative response. Thus, we have proven that long-term exercise is necessary to prevent the liver’s ageing and could be a preventive practice for the putative decline in liver function resulting from ageing. Since we have proven that exercise modifies liver function, it will be important to evaluate whether exercise interferes with drug biotransformation, and to what extent the therapeutic efficacy of specific pharmacological groups is conditioned by physical activity. If this happens, the prescription of physical exercise should consider the triad: exercise/disease/drug. Our results highlight the benefits of lifelong aerobic exercise in preventing early age-related consequences in hepatic mitochondrial and antioxidant function. To the best of our knowledge, this is the first study evaluating 54 weeks of treadmill exercise (program), corresponding to ~30 human years.

## 5. Conclusions

This investigation aimed to assess the impacts of two variables in the hepatic antioxidant and mitochondrial function: the ageing process and a life-long moderate-intensity aerobic exercise plan. This is one of the few studies that has evaluated the impact of the term of the training program in the development of exercise-induced adaptative mechanisms. The results revealed that exercise could alleviate age-related damage regarding mitochondrial function, even though the effects were not as obvious in the antioxidant function. Thus, lifelong exercise seems capable of improving mitochondrial and antioxidant function disruption in the ageing liver. Furthermore, even though exercise intensity is frequently pointed out as crucial for the development of adaptative responses, this study has shown that the duration of the training protocol may be an even more essential parameter for the liver adaptative response.

## Figures and Tables

**Table 1:** Number of animals, ponderal homogeneity index (PH), mortality rate, initial and final animal body weights (g), ponderal gain (PG) and mean relative liver weight for all experimental groups.

Groups	Initial Number of Animals	Final Number of Animals	PH	Mortality Rate (%)	Initial Weight (g)	Final Weight (g)	PG	Mean Relative Liver Weight (g)
NEX 24	10	9	0.63	10	162.7 ± 13.4	471.7 ± 27.8 ^†,&^	0.65 ± 0.03 ^†,&^	0.024 ± 0.03
EX24	10	9	0.56	10	166.3 ± 13.2	406.3 ± 14.5 *^,&^	0.60 ± 0.03 *^,&,#^	0.027 ± 0.01 ^&,#^
NEX54	10	10	0.63	0	152.9 ± 13.4 *^,†,#^	546.2 ± 13.6 *^,†,#^	0.74 ± 0.02 *^,†,#^	0.020 ± 0.05
EX54	10	10	0.55	0	155.6 ± 11.65	439.6 ± 29.7 ^&^	0.65 ± 0.02 ^†,&^	0.023 ± 0.02

Values are means ± SD. * *p* < 0.05 compared with NEX24; ^†^ *p* < 0.05 compared with EX24; ^&^ *p* < 0.05 compared with NEX54; ^#^ *p* < 0.05 compared with EX54; NEX24 (non-exercise young, sacrifice at 24 weeks of training), EX24 (exercise young, sacrifice at 24 weeks of training), NEX54 (non-exercise middle-age, sacrifice at 54 weeks of training), EX54 (exercise middle-age, sacrifice at 54 weeks of training).

**Table 2:** Mean food and water consumption (g) (mean ± SD) at the beginning and at the end of the experimental protocol.

Groups	Initial Food Consumption (g)	Final Food Consumption (g)	Initial Water Consumption (g)	Final Water Consumption (g)
NEX 24	16.4 ± 0.2	17.2 ± 2.8	21.1 ± 1.5	16.3 ± 2.7
EX24	15.5 ± 5.2	19.4 ± 0.8	20.9 ± 0.4	23.8 ± 1.6 ^&,^*
NEX54	14.9 ± 0.6	19.6 ± 2.8	19.6 ± 0.0	16.1 ± 0.4
EX54	15.9 ± 0.9	17.7 ± 0.9	17.3 ± 1.0 ^&,†,^*	20.9 ± 1.6

Values are means ± SD (*n* = 10 for NEX54 and EX54; *n* = 9 for NEX24 and EX24). * *p* < 0.05 compared with NEX24; ^†^ *p* < 0.05 compared with EX24; ^&^ *p* < 0.05 compared with NEX54. NEX24 (non-exercise young, sacrifice at 24 weeks of training), EX24 (exercise young, sacrifice at 24 weeks of training), NEX54 (non-exercise middle-age, sacrifice at 54 weeks of training), EX54 (exercise middle-age, sacrifice at 54 weeks of training).

**Table 3:** Activity of hepatic antioxidant enzymes. Superoxide dismutase (SOD), Catalase (CAT), Glutathione peroxidase (GPx), Glutathione reductase (GR).

	NEX24	EX24	NEX54	EX54
SOD (U.min^−1^.mg^−1^ protein)	2.29 ± 0.28	2.86 ± 0.21 *	2.46 ± 0.31 ^†^	2.79 ± 0.14 *
CAT (mmol H_2_O_2_.min^−1^.mg^−1^ protein)	0.51 ± 0.071	0.58 ± 0.075	0.25 ± 0.030 *^,†^	0.35 ± 0.06 *^,†,&^
GPx (μmol NADPH oxidized.min^−1^.mg^−1^ protein)	429.4 ± 24.0	363.7 ± 40.6 *	381.9 ± 50.9 *	342.4 ± 26.6 *
GR (μmol NADPH oxidized.min^−1^.mg^−1^ protein)	31.44 ± 2.83	25.58 ± 1.71 *	21.89 ± 2.36 *^,†^	20.73 ± 2.13 *^,†^

Values are means ± SD (*n* = 7), with two replicates. * *p* < 0.05 compared with NEX24; ^†^ *p* < 0.05 compared with EX24; ^&^ *p* < 0.05 compared with NEX54; NEX24 (non-exercise young, sacrifice at 24 weeks of training), EX24 (exercise young, sacrifice at 24 weeks of training), NEX54 (non-exercise middle-age, sacrifice at 54 weeks of training), EX54 (exercise middle-age, sacrifice at 54 weeks of training).

**Table 4:** Oxidative stress markers: lipid peroxidation (LPO) and ratio GSH and GSSG.

	NEX24	EX24	NEX54	EX54
LPO (μM MDA.mg^−1^ protein)				
Total Fraction	0.053 ± 0.02	1.16 ± 0.26 *	0.61 ± 0.11 *^,†^	0.53 ± 0.11 *^,†^
Mitochondrial Fraction	0.41 ± 0.14	1.24 ± 0.26 *	0.53 ± 0.06 ^†^	0.33 ± 0.91 ^†^
GSH/GSSG	3.06 ± 0.25	2.68 ± 0.16	3.67 ± 0.91	4.69 ± 1.19 *^,†^

Values are means ± SD (*n* = 7), with two replicates. * *p* < 0.05 compared with NEX24; ^†^ *p* < 0.05 compared with EX24; NEX24 (non-exercise young, sacrifice at 24 weeks of training), EX24 (exercise young, sacrifice at 24 weeks of training), NEX54 (non-exercise middle-age, sacrifice at 54 weeks of training), EX54 (exercise middle-age, sacrifice at 54 weeks of training).

## Data Availability

Not applicable.
